# Three-dimensional semi-automated volumetric assessment of the pulp space of teeth following regenerative dental procedures

**DOI:** 10.1038/s41598-021-01489-8

**Published:** 2021-11-09

**Authors:** Heeresh Shetty, Shishir Shetty, Adesh Kakade, Aditya Shetty, Mohmed Isaqali Karobari, Ajinkya M. Pawar, Anand Marya, Artak Heboyan, Adith Venugopal, The Hanh Nguyen, Dinesh Rokaya

**Affiliations:** 1 Department of Conservative Dentistry and Endodontics, Nair Hospital Dental College, Mumbai, Maharashtra 400008 India; 2 Department of Conservative Dentistry and Endodontics, A. B. Shetty Memorial Institute of Dental Sciences, NITTE (Deemed to be University), Mangalore, India; 3 Department of Pediatric Dentistry, Nair Hospital Dental College, Mumbai, Maharashtra 400008 India; 4 Conservative Dentistry Unit, School of Dental Sciences, Universiti Sains Malaysia, Health Campus, Kubang Kerian, 16150 Kelantan, Malaysia; 5 Department of Orthodontics, University of Puthisastra, Phnom Penh, Cambodia; 6 Department of Prosthodontics, Faculty of Stomatology, Yerevan State Medical University, Str. Koryun 2, 0025 Yerevan, Armenia; 7 Present Address: Department of Orthodontics, Saveetha Dental College, Saveetha Instititute of Medical and Technical Sciences, Chennai, India; 8 VNU School of Medicine and Pharmacy, Vietnam National University, Hanoi, Vietnam; 9 Department of Clinical Dentistry, Walailak University International College of Dentistry, Walailak University, Bangkok, 10400 Thailand

**Keywords:** Cone-beam computed tomography, Pulp conservation

## Abstract

The volumetric change that occurs in the pulp space over time represents a critical measure when it comes to determining the secondary outcomes of regenerative endodontic procedures (REPs). However, to date, only a few studies have investigated the accuracy of the available domain-specialized medical imaging tools with regard to three-dimensional (3D) volumetric assessment. This study sought to compare the accuracy of two different artificial intelligence-based medical imaging programs namely OsiriX MD (v 9.0, Pixmeo SARL, Bernex Switzerland, https://www.osirix-viewer.com) and 3D Slicer (http://www.slicer.org), in terms of estimating the volume of the pulp space following a REP. An Invitro assessment was performed to check the reliability and sensitivity of the two medical imaging programs in use. For the subsequent clinical application, pre- and post-procedure cone beam computed tomography scans of 35 immature permanent teeth with necrotic pulp and periradicular pathosis that had been treated with a cell-homing concept-based REP were processed using the two biomedical DICOM software programs (OsiriX MD and 3D Slicer). The volumetric changes in the teeth’s pulp spaces were assessed using semi-automated techniques in both programs. The data were statistically analyzed using t-tests and paired t-tests (P = 0.05). The pulp space volumes measured using both programs revealed a statistically significant decrease in the pulp space volume following the REP (P < 0.05), with no significant difference being found between the two programs (P > 0.05). The mean decreases in the pulp space volumes measured using OsiriX MD and 3D Slicer were 25.06% ± 19.45% and 26.10% ± 18.90%, respectively. The open-source software (3D Slicer) was found to be as accurate as the commercially available software with regard to the volumetric assessment of the post-REP pulp space. This study was the first to demonstrate the step-by-step application of 3D Slicer, a user-friendly and easily accessible open-source multiplatform software program for the segmentation and volume estimation of the pulp spaces of teeth treated with REPs.

## Introduction

The progressive development of medical imaging techniques has made it possible for an increased number of structures to be assessed and correlated with the morphological alterations associated with disease or treatment. The image segmentation technique and associated tools are commonly used to produce anatomical measurements of lesions or structures. Although the manual assessment of anatomical boundaries by experts remains the benchmark, it is acknowledged as an extremely difficult process due to its labor-intensive and time-consuming nature^1^. Furthermore, as the number of regions of interest (ROI) increases, manually segmented datasets are usually found to contain several small, inaccurately labelled, or detached regions that are difficult to recognize on a two-dimensional display^1^. The semi-automated technique involving the use of label-specific correction tools allows for the rapid identification, navigation, and modification of any small and disconnected erroneous labels within a dataset while still providing valid segmentation results^2^.

Regenerative endodontic procedures (REPs) aim to achieve an organized restoration of the dental pulp and the surrounding structures^3^. In addition, a growing body of evidence supports the ability of REPs to promote root maturation in immature teeth with pulpal necrosis^4–6^. Yet, the use of standard image interpretation systems, which are based on two-dimensional (2D) radiographs, makes it challenging to detect subtle volumetric changes following an REP^7,8^. In these radiographs, any compromise in terms of the geometric configuration results in errors and, therefore, inaccurate readings, which negatively impact the interpretation of the imaging outcomes^9^.

Artificial intelligence (AI)-based imaging algorithms have been the subject of significant research interest in recent years due to their potential to be integrated into cellular and regenerative therapies. However, one major drawback of such technology concerns the possibility of important information being lost when human-interpretable descriptors are involved^2^. Complementing human intelligence with AI could have a significant positive impact on the quality of diagnostic outputs, which suggests the potential for furthering the progress of tailor-made treatment in the field of regenerative endodontics. Of course, all the developed algorithms must ultimately prove their worth in a clinical environment.

Three-dimensional (3D) semi-automated image segmentation by means of label-specific correction tools has previously been demonstrated to be a useful technique for the evaluation of REPs^10^. Although volumetric quantifications obtained from cone beam computed tomography (CBCT) datasets have been utilized for the evaluation of REPs, there remains a clear shortage of well-designed studies applying a standardized quantitative method involving the use of AI-assisted image processing software programs to evaluate the outcomes of REPs. Further, from the clinical and educational perspectives, it is important to recognize that not all clinicians or institutions have access to paid-for software, which indicates the need to validate open-source programs. In light of this need, the present study was designed to quantify and compare the changes in the pulp space volumes of teeth following REPs by means of a semi-automated approach involving two different 3D software programs (i.e., OsiriX MD and 3D Slicer).

## Methodology

### In vitro validation: CBCT segmented volumes (OsiriX MD and 3D Slicer) versus real volumes (laser scanning and water displacement method)

**Figure 1:**
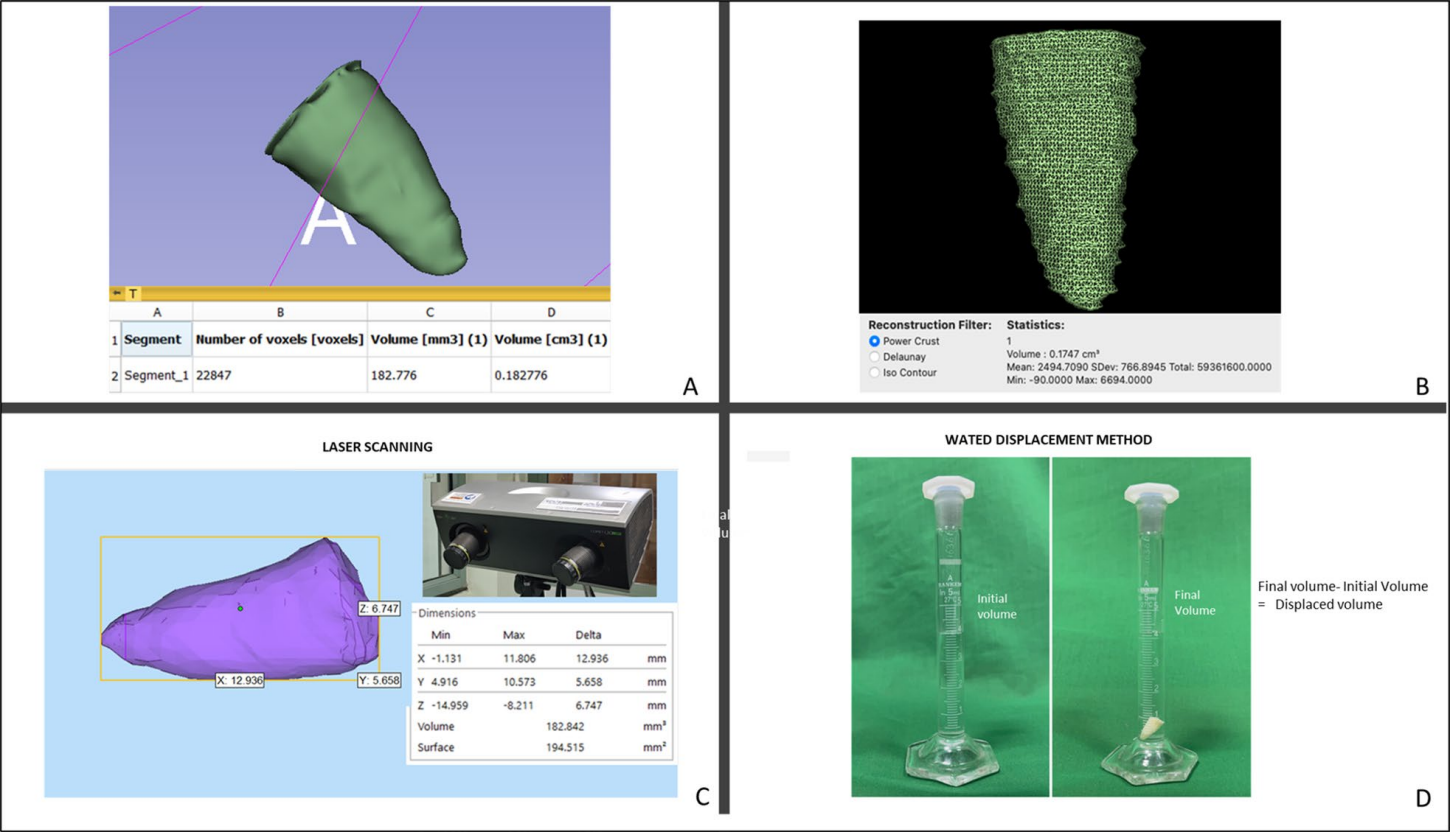
In vitro Validation. Tooth root volume assessment by CBCT segmentation with **(A)** OsiriX MD and **(B)** 3D Slicer software’s. **(C)** Illustrates volumetric analyses of the tooth root in Materialise MiniMagics software after scanning with Steinbichler COMET L3D laser equipment. (D) Illustrates volume estimation of the tooth root by water displacement method.

Five freshly extracted maxillary anterior teeth, cleaned, disinfected, and stored as per the Occupational Safety and Health Administration (OSHA) guidelines were included in the study. The teeth were decoronated at the cemento-enamel junction (CEJ) following which the root canal orifices were sealed with Glass ionomer cement (Fuji IX; GC, Tokyo, Japan). *Radiographic imaging and 3D segmentation:* CBCT scans was obtained for the 5 samples using the CS 9000 3D: Carestream Dental, Atlanta, GA, USA). Each sample was placed in a wax block to prevent any movement during the scanning process. Scanning parameters were fixed at voxel size: 76 µm; FOV: 50 $\times$ 37 mm; tube potential: 70 KVp; tube current: 10 mA; exposure: 10.68 s. The acquired data was exported as a DICOM file into the two imaging platforms. I.e., OsiriX MD (v 9.0, Pixmeo SARL, Bernex Switzerland, https://www.osirix-viewer.com) and 3D Slicer (v 4.8.1, https://www.slicer.org). Tooth segmentation was performed on both the software’s by semi-automated technique with manual refinement on a repeated two-dimensional basis followed by automated computation of the radiographic volume in cubic centimeters (cm^3^). (See Fig. 1A,B) *Laser scan:* The samples were coated with HC 92(Flaw Test Developer, Amol Chemicals, India) to facilitate scanning. The laser scan was performed with the Steinbichler COMET L3D laser equipment (steinbichler, Optotechnik GmbH, Germany). The laser-scanned 3D models were exported as STL format files and imported into Materialise MiniMagics (v 23.5, Materialise, Belgium, https://www.materialise.com/en/software/minimagics) software for quantitative analysis (Fig. 1C).

#### Water displacement method

The volume of each of the samples was measured by the water displacement method in a 5 ml graduated cylinder with 0.1 ml gradations (Rankem, Haryana, India). The cylinder was filled with water at room temperature up to the 4 ml mark. The specimen was immersed completely in the cylinder and the new water level was recorded at the lowest portion of the meniscus. The volume of the specimen was determined by subtracting the initial water volume from the final water volume (Fig. 1D) The measurements obtained were in milliliters (mL), which were converted into cubic centimeters (cm^3^) to unify the readings with the other techniques^11^. (See Table 1).

**Table 1:** In vitro validation. Comparison of CBCT segmented volumes (OsiriX MD and 3 D Slicer) and real volumes (Laser scan and water displacement method).

	OsiriX MD	3D Slicer	Laser Scan	Water Displacement
Mean ± SD (cm^3^)	0.26824 ± 0.059173	0.27600 ± 0.060091	0.2772084 ± 0.060644	0.29000 ± 0.0651920

### Clinical application

Informed consent was obtained from patients or their parents/guardians. The study protocol was approved by the Central ethics committee, Nitte University, Mangalore, India (NU/CEC/Ph.D./-03/2014). The research was carried out in accordance with guidelines and regulations set down in the declaration of Helsinki. This research included CBCT scans of 35 teeth in 28 patients aged 8–38 years with one or more immature permanent teeth with pulp necrosis and apical periodontitis caused either by trauma or caries. Clinical examination focused on the presence of spontaneous pain, swelling, tenderness, and sensitivity to palpation. Cold and electric pulp tests were also performed. Intraoral periapical digital radiographs revealed immature teeth with open apices, showing either wide canals or blunderbuss canals—and in some cases, moderately developed roots but with open apices, along with periapical radiolucency (see Table 2).

**Table 2:** Characteristics of the study population.

Characteristics	Frequency (n)	Percentage (%)
**Age (years)**		
08–18	29	82.7
19–38	06	17.3
Mean 14.26 + 7.031		
**Gender**		
Male	27	77.1
Female	08	22.9
**Tooth**		
Central incisors	32	91.4
Premolars	0303	8.6
**Signs/symptoms**		
Abscess and pain	30	85.7
Cold sensibility and pain	02	5.7
Fistula	03	8.6
**Pulpal status**		
Necrosis	35	100
**Aetiology**		
Caries	03	8.6
Trauma	32	91.4
**Periapical pathology (pre)**		
Present	35	100
**Follow-up (months)**		
18–24	19	54.2
25–39	08	22.8
40–48	08	22.8
Mean 27.14 + 10.318		
**Periapical pathology (post)**		
Present	35	100

### REP protocol

The treatment protocol followed the clinical guidelines proposed by the American Association of Endodontists, as well as the European Society of Endodontology’s position statements^12,13^. The treatments were performed between August 2013 and May 2017 by a single operator using a dental operating microscope (Karl Kaps, Oberkochen, Germany) under a rubber dam.

In the first appointment, after a local anaesthetic with adrenaline was administered, an endodontic access cavity was made, followed by disinfection of the root canal using 1.5% NaOCl. The canal was then dried by aspiration (without air blow) and the application of sterile paper points, followed by the application of 1 mg/mL triple antibiotic paste. The tooth was sealed with an intermediate restorative material (IRM) (Dentsply International, Milford, DE, USA) and recalled after two to three weeks. In the next appointment, the response to the initial treatment was assessed. If clinical signs and symptoms continued to persist, the same treatment protocol was repeated.

If the tooth was symptom-free and the canal was found to be dry, the REP was carried out as follows. The root canal was re-entered and irrigated with a copious amount of 1.5% NaOCl to remove the antibiotic dressing material and all debris. The root canal dentin was conditioned using EDTA 17% followed by laceration of the apical tissue with a sterile 23-gauge needle to induce bleeding into the root canal so that the canal would fill with blood to the level of the cementoenamel junction (CEJ). A sterile, biodegradable collagen plug (CollaPlug; Zimmer Biomet, Warsaw, IN, USA) was placed over the blood clot, which served as a scaffold. A coronal seal, made from either white mineral trioxide aggregate (MTA) (ProRoot; Dentsply Sirona, Ballaigues, Switzerland) or Biodentine (Septodont, aint-Maur-des-Fosses, France), was placed as close as possible to the level of the CEJ. Then, the tooth was sealed with glass ionomer cement (Fuji IX; GC, Tokyo, Japan), followed by a composite restoration (Filtek Z250/Z350; 3 M ESPE, Saint Paul, MN, USA). At the end of the procedure, a CBCT scan was obtained (CS 9000 3D; voxel size: 76 µm; FOV: 50 $\times$ 37 mm; tube potential: 70 KVp; tube current: 10 mA; exposure: 10.68 s; Carestream Dental, Atlanta, GA, USA).

### Follow-up

Patients were recalled at six-month intervals after REP. At each recall session, a digital periapical radiograph was taken, and clinical tests were performed. Clinical success was defined as a tooth that survived and did not require another endodontic intervention during the recall period^14^. Once significant radiographic changes were evident during the observation time, a final follow-up CBCT scan was taken (CS 9000 3D; voxel size: 76 µm; FOV: 50 $\times$ 37 mm; tube potential: 70 KVp; tube current: 10 mA; exposure: 10.68 s; Carestream Dental, Atlanta, GA, USA).

### Volume measurements

**Figure 2:**
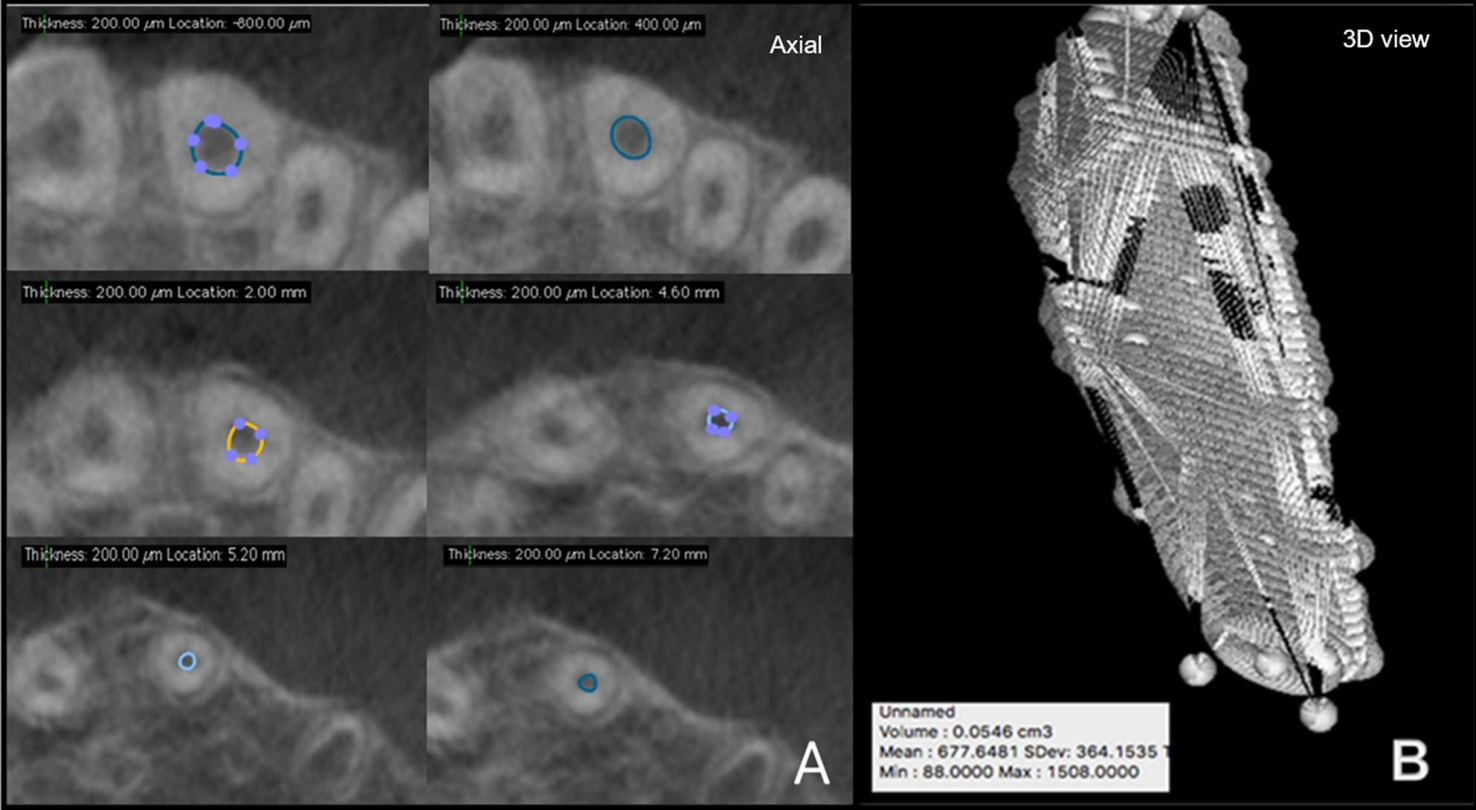
Pulp space volume assessment using OsiriX MD (**A)** illustrates the ROI in various axial slices and **(B)** reconstructed 3D model along with the volume statistics.

The initial and the final CBCT datasets were processed to quantify changes in the post-REPT pulp space volume using two biomedical DICOM software programs: OsiriX MD (v 9.0, Pixmeo SARL, Bernex Switzerland, https://www.osirix-viewer.com/osirix/osirix-md/) and 3D Slicer (v 4.8.1, https://download.slicer.org/).The pulp space volume was measured by an endodontist who was proficient in the use of OsiriX MD. The “closed polygon selection” tool, found under the ROI tool button, was used to trace the boundary of the pulp space on alternate slices (axial images, slice thickness: 0.3 mm) from a fixed coronal reference point (i.e., the end of the coronal seal) to the apex of the root. After outlining only half of the slices between the two reference points, the missing ROI can be generated using the “generate missing ROIs” tool under the ROI dropdown menu. The “Grow Region (2D/3D Segmentation)” algorithm facilitates this automated process based on the differences in Hounsfield units between the pulp space and the surrounding hard tissues. The automated outlines are manually adjusted using the “closed polygon selection” tool and the “repulsor tool” to refine the ROI (see Fig. 2A). After collecting all the ROI within one series, the “ROI volume” tool automatically calculates the volume by multiplying surface and slice thickness and then adds up the individual slice volumes to construct a 3D model (Fig. 2B). The Macintosh operating system was used (Mac OS; Intel Core i5, 1.8 GHz, 4 GB RAM).

The volume measurements using the 3D Slicer software were taken by an experienced endodontist with prior experience of the software and their plug-ins. The Macintosh operating system was used (Mac OS; Intel Core i5, 1.8 GHz, 4 GB RAM). Pulp space was segmented using the GrowCut technique and morphological operations such as erosion, dilation, and island removal.

**Figure 3:**
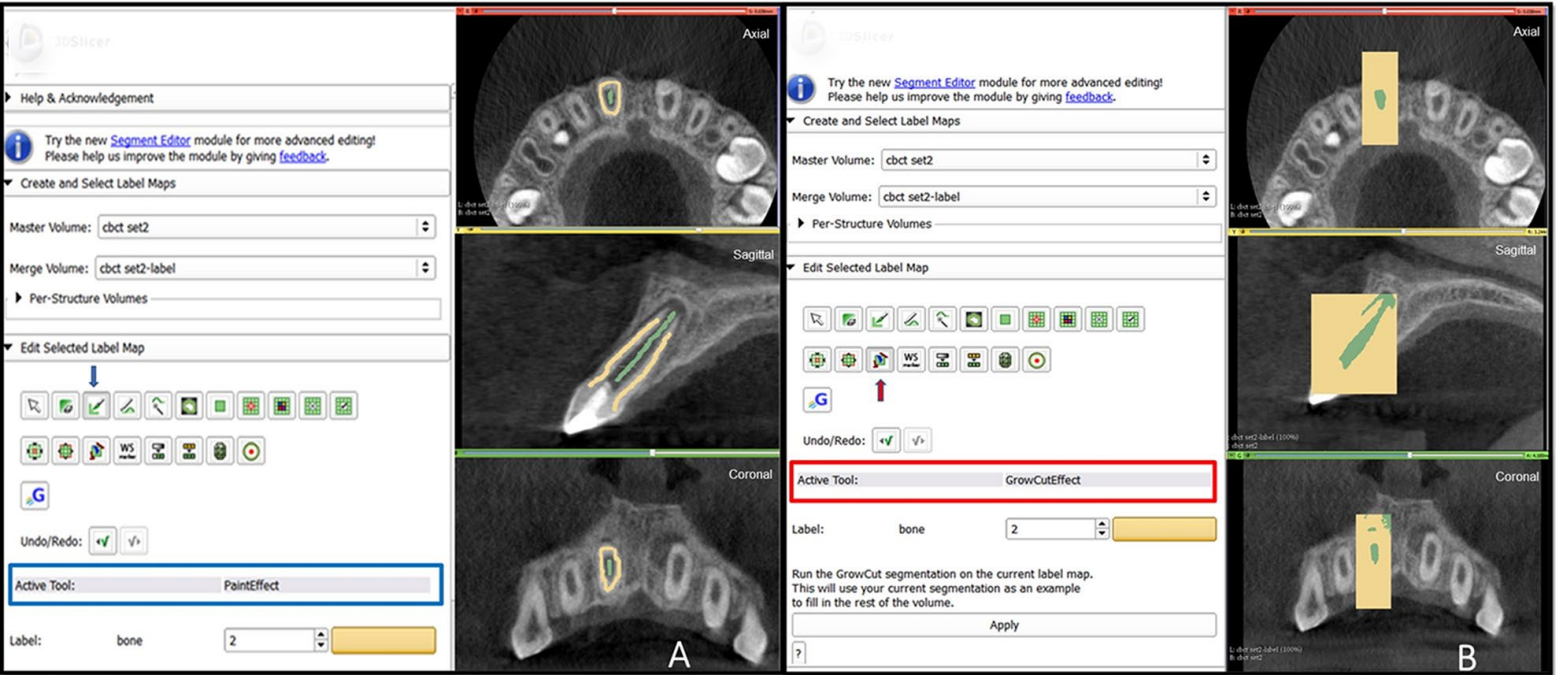
Grow Cut technique in 3D Slicer (**A)** initialization of an area inside and outside the ROI using paint effect (blue arrow). **(B)** automatic competing region- growing using GrowCut effect (red arrow).

**Figure 4:**
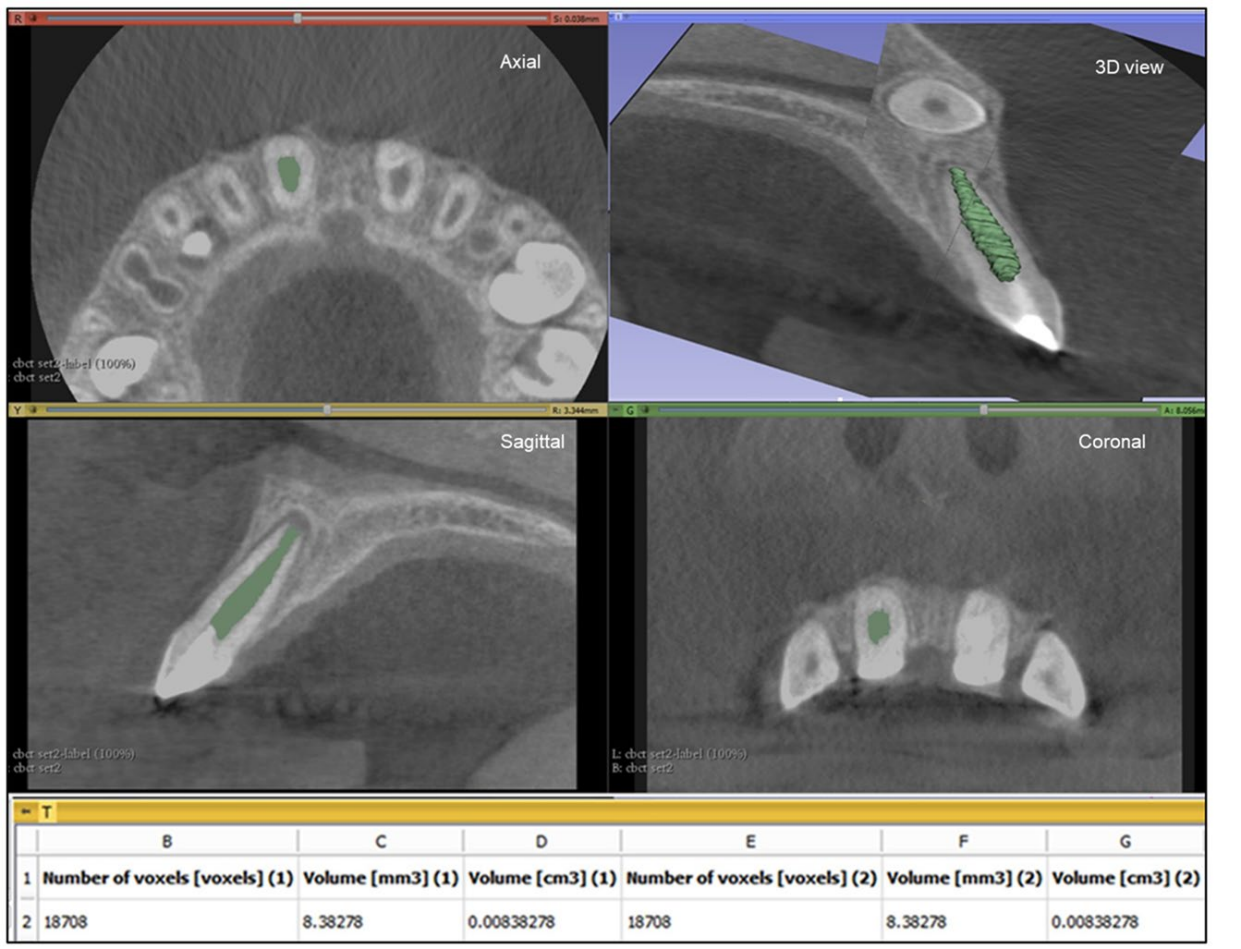
Pulp space volume assessment using 3D Slicer: illustrates the ROI in the axial, saggital and coronal slicing as well as the reconstructed 3D model with the calculated volume.

The following workflow was performed for pulp space segmentation: (a) the CBCT dataset was loaded into 3D Slicer, b) identification of a region inside the pulp space preceded by a stroke with a brush size of around 0.5 cm beyond the pulp space (see Fig. 3A), (c) automatic competing region-growing was performed using GrowCut (see Fig. 3B), (d) editing tools were used for manual refinement after visual inspection of results (qualitative assessment), and (e) “quantification” module was used to extract the pulp space volume (see Fig. 4).

#### 3-dimensional superimposition

**Figure 5:**
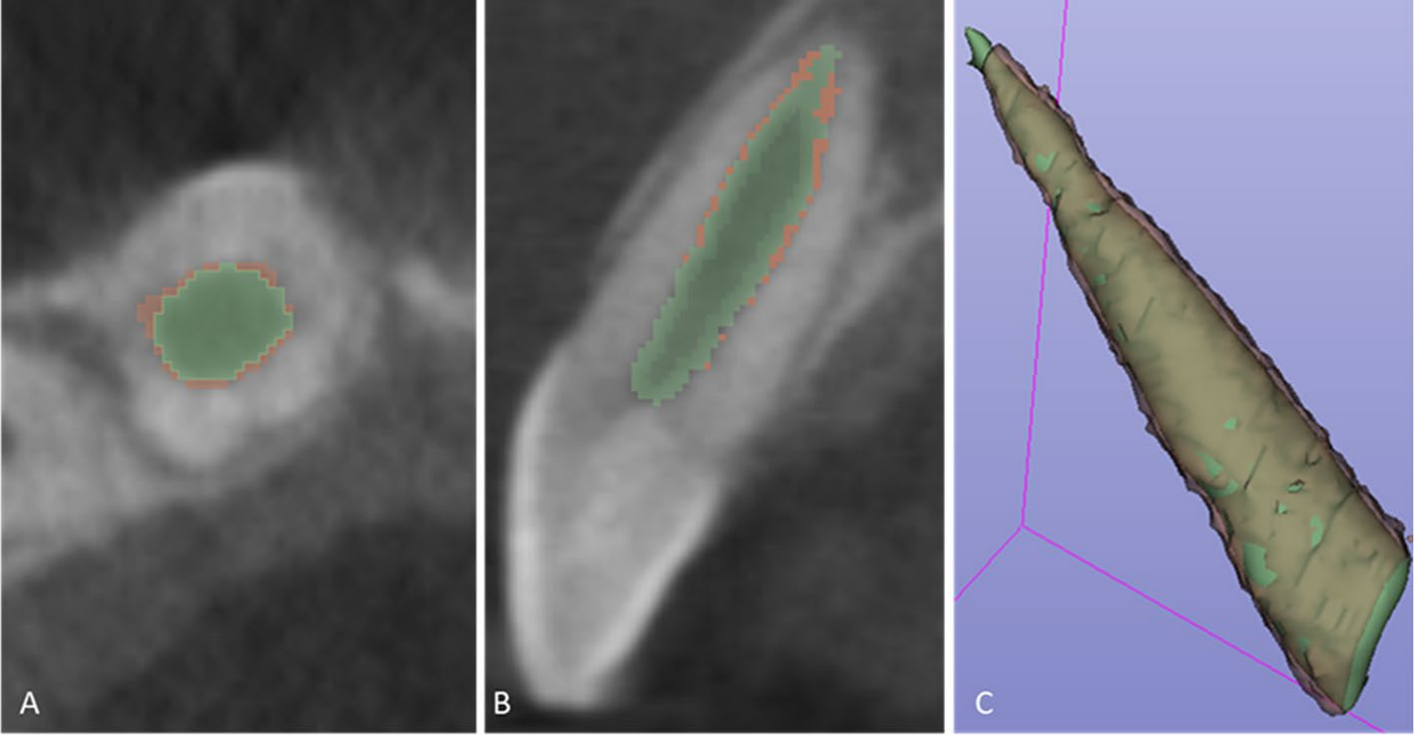
Voxel-based superimposition. Axial slice (**A**), sagittal slice (**B**) and 3d reconstruction (**C**) showing post pulp volume segmentation (green) superimposed over the pre pulp volume segmentation (brown).

Voxel- based superimposition was carried out using the CM Freg extension of the 3D slicer. Cranial based registration of the pre and post pulp space volume segmentations were done under the growing modules of CMF reg in 3D slicer. During the superimposition (registration), the post pulp volume segmentation is moved and automatically superimposed on a static pre pulp volume segmentation creating a registered surface model. (See Fig. 5A–C) *Digital subtraction*: The change in volume post REP was alternatively analyzed by “pixelwise subtraction” of the post pulp volume from the pre pulp volume using the “Subtract scalar volume” parameter under the “Filtering” module of the 3D slicer. The resultant volume was quantified using the “segment statistics parameter” under the “quantification” module.

Both evaluators conducted the measurements separately and were blinded to each other’s results. A separate dataset of five patients was evaluated to assess intra-observer variability, producing a mean Dice similarity coefficient of 90.45 ± 2.71 (OsiriX MD) and 93.16 ± 2.56 (3D Slicer). Because intra-observer reliability was high for both evaluators, the measurements were performed only once.

The results were generated in millimeter and centimeter cube units and the data presented in percentage (see Table 3).

**Table 3:** Patient data sheet.

Age/Sex	Tooth	Pre volume OsiriX MD (Cm^3^)	Time for volume estimation (Min)	Post volume OsiriX MD (Cm^3^)	Time for volume estimation (Min)	Change in volume OsiriX MD (Cm^3^)	Change in volume OsiriX MD (%)	Pre volume 3D Slicer (Cm^3^)	Time for volume estimation (Min)	Post volume 3D Slicer (Cm^3^)	Time for volume estimation (Min)	Change in volume 3D Slicer (Cm^3^)	Change in volume 3D Slicer (%)
9/M	11	0.0386	14	0.0297	14	0.0089	23.0569	0.0361	9	0.0267	9	0.0094	26.0387
13/M	35	0.0252	13	0.0186	13	0.0066	26.1904	0.0241	9	0.0169	9	0.0072	29.8755
9/M	11	0.0276	14	0.0221	14	0.0055	19.9275	0.0279	9	0.0233	9	0.0046	16.4874
18/F	21	0.0172	14	0.0159	13	0.0013	07.5581	0.0181	9	0.0162	9	0.0019	10.4972
18/M	11	0.0422	13	0.0383	12	0.0039	09.2417	0.0411	9	0.0375	9	0.0036	08.7591
18/M	21	0.0365	13	0.0220	13	0.0145	39.7260	0.0361	9	0.0209	9	0.0152	42.1052
10/M	11	0.0282	13	0.0009	19	0.0272	96.5936	0.0251	9	0.0008	13	0.0242	96.4537
10/M	21	0.0262	13	0.0191	13	0.0071	27.0992	0.0269	9	0.0189	9	0.0080	29.7397
18/F	11	0.0473	12	0.0334	13	0.0139	29.3868	0.0473	9	0.0301	9	0.0172	36.3636
33/M	21	0.0546	14	0.0442	14	0.0104	19.0476	0.0510	9	0.0396	9	0.0114	22.3529
38/F	11	0.0295	13	0.0212	13	0.0083	28.1355	0.0273	8	0.0203	9	0.0070	25.6410
13/M	21	0.0235	13	0.0209	12	0.0026	11.0638	0.0237	9	0.0197	9	0.0040	16.8776
20/M	11	0.0543	13	0.0441	12	0.0102	18.7845	0.0523	9	0.0437	9	0.0086	16.4435
20/M	21	0.0710	13	0.0657	13	0.0053	07.4647	0.0717	9	0.0651	9	0.0066	09.2050
8/F	21	0.0206	14	0.0158	13	0.0048	23.3009	0.0209	9	0.0155	9	0.0054	25.8373
11/M	11	0.0214	14	0.0070	14	0.0143	67.2102	0.0197	9	0.0071	9	0.0125	63.8223
11/F	45	0.0269	13	0.0239	13	0.0030	11.1524	0.0261	8	0.0235	9	0.0026	09.9616
10/M	21	0.0262	13	0.0131	14	0.0131	50.0000	0.0249	9	0.0121	9	0.0128	51.4056
19/M	21	0.0098	15	0.0096	14	0.0001	01.7017	0.0083	9	0.0080	9	0.0002	2.78464
13/M	11	0.0361	13	0.0282	13	0.0079	21.8836	0.0347	9	0.0252	9	0.0095	27.3775
13/M	21	0.0315	13	0.0275	12	0.0040	12.6984	0.0309	9	0.0268	9	0.0041	13.2686
13/M	21	0.0143	13	0.0137	13	0.0006	04.1958	0.0147	9	0.0131	9	0.0016	10.8843
20/M	11	0.0487	13	0.0385	13	0.0102	20.9445	0.0478	9	0.0379	9	0.0099	20.7246
10/M	11	0.0181	12	0.0141	13	0.0040	22.0994	0.0172	9	0.0135	9	0.0037	21.5116
10/M	21	0.0161	13	0.0147	13	0.0014	08.6956	0.0153	9	0.0129	9	0.0024	15.6862
9/M	21	0.0253	13	0.0226	13	0.0027	10.6719	0.0242	9	0.0221	9	0.0021	08.6776
10/M	11	0.0362	13	0.0257	13	0.0105	29.0055	0.0370	9	0.0261	9	0.0109	29.4594
10/M	21	0.0387	13	0.0288	12	0.0099	25.5814	0.0365	9	0.0291	9	0.0074	20.2739
12/F	34	0.0374	12	0.0277	12	0.0097	25.9358	0.0381	9	0.0283	9	0.0098	25.7217
10/M	21	0.0283	14	0.0108	13	0.0175	61.8374	0.0269	8	0.0101	9	0.0168	62.4535
11/M	11	0.0141	13	0.0081	13	0.0059	41.9007	0.0151	9	0.0092	9	0.0058	38.6006
11/M	21	0.0093	13	0.0085	13	0.0007	07.8593	0.0089	9	0.0081	9	0.0008	09.3645
18/F	21	0.0289	13	0.0214	12	0.0075	25.9515	0.0292	8	0.0211	9	0.0081	27.7397
10/F	21	0.0219	13	0.0190	12	0.0029	13.2420	0.0207	9	0.0179	9	0.0028	13.5265
9/M	21	0.0361	13	0.0260	13	0.0101	27.9778	0.0386	9	0.0279	9	0.0107	27.7202

*Cm*^*3*^ cubic centimeters, *%* Percentage, *Min* minutes.

### Data analysis

Statistical analysis of the data was performed using SPSSS (v 21.0, IBM). For In vitro validation a comparison between the CBCT segmented volumes (OsiriX MD and 3 D Slicer) and real volumes (laser scan and water displacement method) for the five samples was undertaken to check the sensitivity of the two software programs. Correlation between groups was performed to determine the reliability of the recorded volumes. Data was analyzed using Bland Altman method and Intraclass correlation coefficient.

For Clinical data analysis, normality of numerical data was assessed using the Shapiro–Wilk test and parametric tests were used for comparisons. Intergroup comparisons were made using the t-test. The intragroup comparison was made using paired t-test. A P-value < 0.05 was considered as significant, keeping α error at 5% and β error at 20%, thus giving power to the study as 80%.

## Results

### In vitro validation

**Figure 6:**
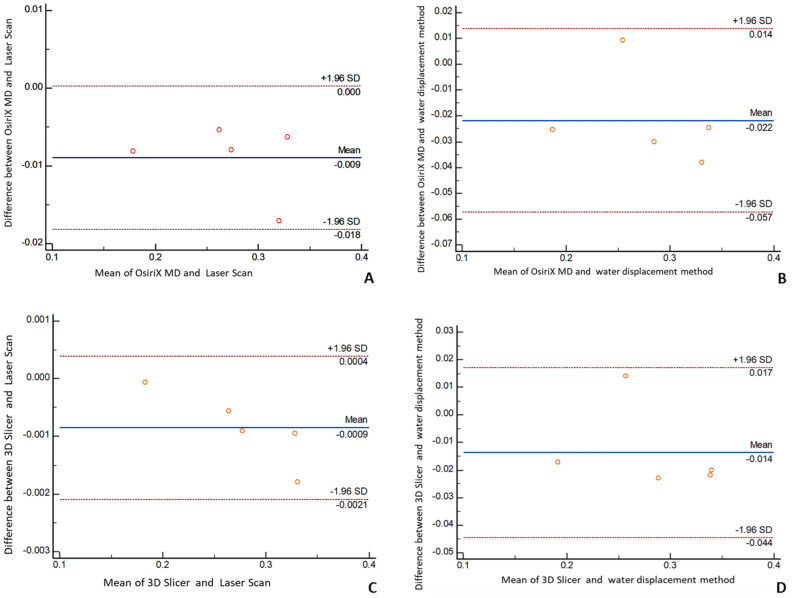
Invitro validation. Bland–Altman Plot comparison between **(A)** CBCT segmented volumes from OsiriX MD and real volumes from Laser scans. **(B)** CBCT segmented volumes from OsiriX MD and real volumes from water displacement method **(C)** CBCT segmented volumes from 3D Slicer and real volumes from Laser scans. **(D)** CBCT segmented volumes from 3D Slicer and real volumes from water displacement method. The difference between the measurements is plotted against their mean.

The Bland and Altman plots and the Intraclass correlation coefficient presented an absolute agreement between CBCT segmented volumes (OsiriX MD and 3D Slicer) and real volumes (laser scan and water displacement method). (See Fig. 6, Table 4).

**Table 4:** In vitro validation.

Groups	Intraclass correlation	95% confidence interval
OsiriX MD & Laser Scan	0.9865	0.3418–0.9988
OsiriX MD & Water displacement method	0.9096	0.1476–0.9907
3D Slicer & Laser Scan	0.9999	0.9942–1.0000
3D slicer & Water displacement method	0.9521	0.5675–0.9950

Intraclass correlation coefficient between groups.

### Clinical outcome

**Figure 7:**
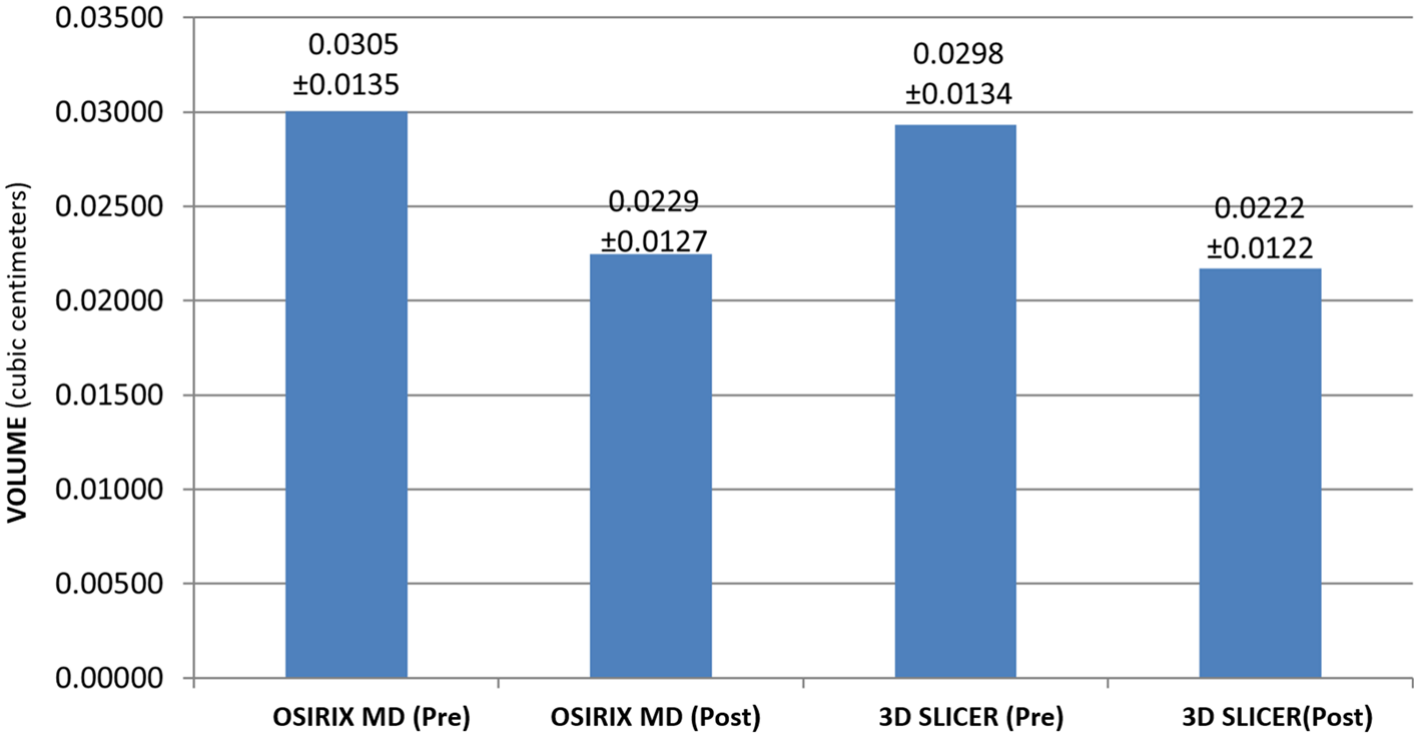
Bar diagram depicting the decrease in volume (mean) calculated with OsiriX MD and 3D Slicer based on the initial (pre) and the final (post) CBCT scans.

The pulp space volume measured by two different medical imaging programs (OsiriX MD and 3D Slicer) showed a statistically significant decrease in post REP pulp space volume (See Fig. 7). The mean decrease in pulp space volume with OsiriX MD was 25.06% ± 19.45%, and 3D Slicer was 26.10.% ± 18.90%. However, no significant difference was found between OsiriX MD & 3D Slicer values (p > 0.05). The mean time taken for volumetric analyses in a patient was 13.14 ± 0.22 for OsiriX MD and 9 ± 0.12 min with 3D Slicer which was statistically significant.

## Discussion

Advancements in 3D imaging tools that minimize human effort have yielded images regarding pathological changes with greater sensitivity and improved anatomical resolution, which has facilitated clinical research and clinical practice. Further, the development of 3D medical imaging software programs allows the analysis and quantitative interpretation of the obtained data, aiding in the identification of small and previously undetectable quantitative variations. This improves the precision concerning diagnosing and evaluating individual responses to a given treatment. It has been established that assessing 3D anatomical structures using their 2D equivalents may lead to errors^15^. To overcome this problem, it is recommended to use concepts of geometric correction and 3D multiplanar reconstructions (MPRs)^15,16^.

Once imaging data are acquired, the next step is image segmentation, which refers to the delineation of the desired anatomy, or ROI. Discriminating the anatomy of interest from the surrounding tissues often requires expertise and time, and sufficient knowledge of specialized software to perform the segmentation. Time taken for segmentation may vary significantly depending upon the ROI. Some software programs offer algorithms and protocols that are tailored to define certain anatomical regions more efficiently. No standardized approach to image segmentation currently exists, and the segmentation process can be automated or manual. However, many workflows promote a semi-automated approach similar to the one discussed in the present study. This is so because a fully automated approach often fails to match human assessment, leading to procedural errors that reflect on the final measurements, especially with regard to the low-contrast images produced by CBCT^17^.

In addition to periapical healing, the primary outcome measures of REP include the elimination of clinical signs and symptoms and the increased thickness of root canal walls and root length. All the latter outcomes are assessed radiographically using 2D or 3D imaging^18,19^. While this concern was addressed previously in our work^14^ and those of others^9,10^, a critical factor that influences radiographic outcomes pertains to the method by which the radiographic images are analyzed. Therefore, this study focused on applying an AI-assisted imaging technology that enhances tissue-based detection and segmentation^20^ as a secondary analysis for our recently published clinical study^14^. The results of this study will further improve the accuracy of three-dimensionally engineered scaffolds and play a significant role in performing cell-based regenerative endodontic procedures^21^.

OsiriX MD is a medical image processing application for Mac that runs on a 64-bit platform fully compliant with the DICOM standard for image communication and image file formats. It is an FDA-approved 510 k class II medical device per the US Food and Drug Regulation CFR21 part 820. OsiriX MD has been utilized to analyze the pulp space volume post REP^22^. The advantage with this software is that there is no need to outline the pulp space boundaries in every slice because it interpolates the ROI for the missed slices and computes the volume. On the other hand, 3D Slicer is an open-source software platform for medical image informatics, image processing, and 3D visualization. It works across all operating platforms, such as Linux, Mac OS, and Windows^23^. The front end of the algorithm is simple to use, requiring no further inputs from the user besides the painted strokes on the desired ROI (Pulp Space) and background using different colors. However, at the back end of the algorithm, once segmentation is computed, user-defined morphological operations such as erosion, dilation, and island removal are required for an efficient segmentation of the ROI. This step may require the user to get accustomed to the variety of tools and modules available within the software. Although the use of 3D Slicer software is not restricted, the FDA has still not approved it for clinical use.

Typically, proprietary DICOM imaging software programs are expensive and their accessibility to the general clinician is limited. Therefore, the purpose of this study was to evaluate whether an open-source software (3D Slicer) approach would be practical and efficient for the volumetric analyses of pulp space post REP when compared to that of proprietary software (OsiriX MD).

The results of this work demonstrated that the mean decrease in volume after REP was 7.62 mm^3^ with OsiriX MD and 7.685 mm^3^ with 3D Slicer. Statistically, there was no significant difference in the mean change in volumes calculated. This result can be attributed to the dedicated tools within these software packages that allow the rapid, automatic outline and measurement of the ROI. Specifically, the operator is required only to detect the coronal and apical extent (reference point), thereby minimizing observer variations when analyzing 3D images in various planes^24^.

Except for a case report and one case series, there are currently no studies in literature that report the volumetric analyses of the teeth after REP^10,22^. Mostafa EzEldeen et al. conducted a similar study using the two-step livewire, semiautomatic user-guided 3D active contour segmentation technique with MeVisLab (MeVis Research, Bremen, Germany) software. The mean change in volume after REP was reported to be 27.92 mm^3^ in the five-case series^10^. This difference in outcome compared to our study may be attributed to the larger number of cases and other predisposing factors, such as the etiology (trauma) and periapical pathosis in the present study population^14^.

The reduction in volume of pulp space post REP was apparently due to the intra canal deposition of cementum or bone-like tissues. This assumption is based on various reported histological findings in immature teeth with apical periodontitis treated with REP^22,25–27^. However, the nature of the tissues formed or the influence of predisposing factors (i.e., trauma, periapical pathosis) has not been discussed nor was it in the scope of the present study, which mainly focused on the quantitative efficiency of the two software programs. Although an attempt was made to quantify the hard tissue deposition on the canal walls post REP, we were unsuccessful in delineating its boundaries. This outcome may be due to some of the limitations of CBCT imaging, such as a low contrast, background noise, limited correlation with Hounsfield units, along with a small area of tissue to be assessed. All these reasons made it difficult to determine the precise location and quantification of the hard tissue formed post REP^28^. However, with 3D Slicer we were able to superimpose the post REP segmented pulp space volume over its pre-operative counterpart to assess the change in volume. The resultant change in volume calculated from “superimposition and digital subtraction” corresponded with the values of the conventional technique used for analyses in the present study.

In terms of efficiency, the process with OsiriX MD was more time consuming compared to the grow cut technique in 3D Slicer. In OsiriX MD, multiple points outlining the diameter of the pulp space must be marked manually on the selected slices until the entire perimeter is defined, thereby increasing its working time.

## Conclusion

To the best of our knowledge, there has been no study comparing the efficiency of OsiriX MD and 3D Slicer for volumetric analyzes of the pulp space post REP. Though a statistically significant difference in volume was recorded post REP, the present study demonstrated that both programs can be used with similar results. The open-source software, i.e., 3D Slicer, used for volumetric analyses in the present study seems to offer the advantages of being significantly faster and requiring lesser user interaction, thereby showing potential for end-user application in assessing outcomes of REP. Despite the steep learning curve associated with newer medical imaging programs, it is necessary for the adoption of these techniques and technologies into practice, the benefits of which outweigh the efforts.

## Supplementary Information

Supplementary Information.
