# Supplementary material for: Three-dimensional semi-automated volumetric assessment of the pulp space of teeth following regenerative dental procedures
Source: Sci Rep. 2021 Nov 9;11:21914. doi: 10.1038/s41598-021-01489-8 (PMC8578625; doi:10.1038/s41598-021-01489-8)
Supplement: Supplementary file 1 — Supplementary Information. [file 41598_2021_1489_MOESM1_ESM.docx]

Table 1: Volumetric estimation details obtained using OsiriX and 3D Slicer

| **Age/Sex** | **Tooth** | **Pre volume OsiriX MD (Cm^3^)** | **Time for volume estimation (Min)** | **Post volume OsiriX MD (Cm^3^)** | **Time for volume estimation (Min)** | **Change in volume OsiriX MD (Cm^3^)** | **Change in volume OsiriX MD (%)** | **Pre volume 3D Slicer (Cm^3^)** | **Time for volume estimation (Min)** | **Post volume 3D Slicer (Cm^3^)** | **Time for volume estimation (Min)** | **Change in volume 3D Slicer (Cm^3^)** | **Change in volume 3D Slicer (%)** |
| --- | --- | --- | --- | --- | --- | --- | --- | --- | --- | --- | --- | --- | --- |
| 9/M | 11 | 0.0386 | 14 | 0.0297 | 14 | 0.0089 | 23.05699 | 0.0361 | 9 | 0.0267 | 9 | 0.0094 | 26.03878 |
| 13/M | 35 | 0.0252 | 13 | 0.0186 | 13 | 0.0066 | 26.19048 | 0.0241 | 9 | 0.0169 | 9 | 0.0072 | 29.87552 |
| 9/M | 11 | 0.0276 | 14 | 0.0221 | 14 | 0.0055 | 19.92754 | 0.0279 | 9 | 0.0233 | 9 | 0.0046 | 16.48746 |
| 18/F | 21 | 0.0172 | 14 | 0.0159 | 13 | 0.0013 | 7.55814 | 0.0181 | 9 | 0.0162 | 9 | 0.0019 | 10.49724 |
| 18/M | 11 | 0.0422 | 13 | 0.0383 | 12 | 0.0039 | 9.241706 | 0.0411 | 9 | 0.0375 | 9 | 0.0036 | 8.759124 |
| 18/M | 21 | 0.0365 | 13 | 0.022 | 13 | 0.0145 | 39.72603 | 0.0361 | 9 | 0.0209 | 9 | 0.0152 | 42.10526 |
| 10/M | 11 | 0.0282 | 13 | 0.000961 | 19 | 0.027239 | 96.59362 | 0.0251 | 9 | 0.00089 | 13 | 0.02421 | 96.45378 |
| 10/M | 21 | 0.0262 | 13 | 0.0191 | 13 | 0.0071 | 27.09924 | 0.0269 | 9 | 0.0189 | 9 | 0.008 | 29.73978 |
| 18/F | 11 | 0.0473 | 12 | 0.0334 | 13 | 0.0139 | 29.38689 | 0.0473 | 9 | 0.0301 | 9 | 0.0172 | 36.36364 |
| 33/M | 21 | 0.0546 | 14 | 0.0442 | 14 | 0.0104 | 19.04762 | 0.051 | 9 | 0.0396 | 9 | 0.0114 | 22.35294 |
| 38/F | 11 | 0.0295 | 13 | 0.0212 | 13 | 0.0083 | 28.13559 | 0.0273 | 8 | 0.0203 | 9 | 0.007 | 25.64103 |
| 13/M | 21 | 0.0235 | 13 | 0.0209 | 12 | 0.0026 | 11.06383 | 0.0237 | 9 | 0.0197 | 9 | 0.004 | 16.87764 |
| 20/M | 11 | 0.0543 | 13 | 0.0441 | 12 | 0.0102 | 18.78453 | 0.0523 | 9 | 0.0437 | 9 | 0.0086 | 16.44359 |
| 20/M | 21 | 0.071 | 13 | 0.0657 | 13 | 0.0053 | 7.464789 | 0.0717 | 9 | 0.0651 | 9 | 0.0066 | 9.205021 |
| 8/F | 21 | 0.0206 | 14 | 0.0158 | 13 | 0.0048 | 23.30097 | 0.0209 | 9 | 0.0155 | 9 | 0.0054 | 25.83732 |
| 11/M | 11 | 0.0214 | 14 | 0.007017 | 14 | 0.014383 | 67.21028 | 0.0197 | 9 | 0.007127 | 9 | 0.012573 | 63.82234 |
| 11/F | 45 | 0.0269 | 13 | 0.0239 | 13 | 0.003 | 11.15242 | 0.0261 | 8 | 0.0235 | 9 | 0.0026 | 9.961686 |
| 10/M | 21 | 0.0262 | 13 | 0.0131 | 14 | 0.0131 | 50 | 0.0249 | 9 | 0.0121 | 9 | 0.0128 | 51.40562 |
| 19/M | 21 | 0.009849 | 15 | 0.009681 | 14 | 0.000168 | 1.701782 | 0.008328 | 9 | 0.008096 | 9 | 0.000232 | 2.784649 |
| 13/M | 11 | 0.0361 | 13 | 0.0282 | 13 | 0.0079 | 21.88366 | 0.0347 | 9 | 0.0252 | 9 | 0.0095 | 27.37752 |
| 13/M | 21 | 0.0315 | 13 | 0.0275 | 12 | 0.004 | 12.69841 | 0.0309 | 9 | 0.0268 | 9 | 0.0041 | 13.26861 |
| 13/M | 21 | 0.0143 | 13 | 0.0137 | 13 | 0.0006 | 4.195804 | 0.0147 | 9 | 0.0131 | 9 | 0.0016 | 10.88435 |
| 20/M | 11 | 0.0487 | 13 | 0.0385 | 13 | 0.0102 | 20.94456 | 0.047824 | 9 | 0.037912 | 9 | 0.009911 | 20.72466 |
| 10/M | 11 | 0.0181 | 12 | 0.0141 | 13 | 0.004 | 22.09945 | 0.0172 | 9 | 0.0135 | 9 | 0.0037 | 21.51163 |
| 10/M | 21 | 0.0161 | 13 | 0.0147 | 13 | 0.0014 | 8.695652 | 0.0153 | 9 | 0.0129 | 9 | 0.0024 | 15.68627 |
| 9/M | 21 | 0.0253 | 13 | 0.0226 | 13 | 0.0027 | 10.67194 | 0.0242 | 9 | 0.0221 | 9 | 0.0021 | 8.677686 |
| 10/M | 11 | 0.0362 | 13 | 0.0257 | 13 | 0.0105 | 29.00552 | 0.037 | 9 | 0.0261 | 9 | 0.0109 | 29.45946 |
| 10/M | 21 | 0.0387 | 13 | 0.0288 | 12 | 0.0099 | 25.5814 | 0.0365 | 9 | 0.0291 | 9 | 0.0074 | 20.27397 |
| 12/F | 34 | 0.0374 | 12 | 0.0277 | 12 | 0.0097 | 25.93583 | 0.0381 | 9 | 0.0283 | 9 | 0.0098 | 25.72178 |
| 10/M | 21 | 0.0283 | 14 | 0.0108 | 13 | 0.0175 | 61.83746 | 0.0269 | 8 | 0.0101 | 9 | 0.0168 | 62.45353 |
| 11/M | 11 | 0.0141 | 13 | 0.008192 | 13 | 0.005908 | 41.90071 | 0.0151 | 9 | 0.009271 | 9 | 0.005829 | 38.60066 |
| 11/M | 21 | 0.009306 | 13 | 0.008575 | 13 | 0.000731 | 7.859361 | 0.008938 | 9 | 0.008101 | 9 | 0.000837 | 9.364511 |
| 18/F | 21 | 0.0289 | 13 | 0.0214 | 12 | 0.0075 | 25.95156 | 0.0292 | 8 | 0.0211 | 9 | 0.0081 | 27.73973 |
| 10/F | 21 | 0.0219 | 13 | 0.019 | 12 | 0.0029 | 13.24201 | 0.0207 | 9 | 0.0179 | 9 | 0.0028 | 13.52657 |
| 9/M | 21 | 0.0361 | 13 | 0.026 | 13 | 0.0101 | 27.97784 | 0.0386 | 9 | 0.0279 | 9 | 0.0107 | 27.72021 |

**Cm^3 -^ cubic centimetres: %- Percentage: Min- Minutes.**
